# Neuron-Specific Enolase as a Predictor of Neurologic Outcomes in Extracorporeal Cardiopulmonary Resuscitation Patients

**DOI:** 10.3390/jcm13144135

**Published:** 2024-07-15

**Authors:** Yong Ho Jeong, Suk Kyung Lim, Yongil Cho, Yun Jin Kim, Hyo Jun Jang, Yang Hyun Cho, Yonghoon Shin, Jae Seung Jung, Jin Kook Kang, Sung-Min Cho, Jun Ho Lee

**Affiliations:** 1 Department of Thoracic and Cardiovascular Surgery, Gangnam Severance Hospital, Yonsei University College of Medicine, Seoul 06273, Republic of Korea; fauntleroy@naver.com; 2 Department of Thoracic and Cardiovascular Surgery, Samsung Medical Center, Sungkyunkwan University College of Medicine, Seoul 06351, Republic of Korea; seokkyunglim@gmail.com (S.K.L.); mdcho95@gmail.com (Y.H.C.); 3 Department of Emergency Medicine, Hanyang University Seoul Hospital, Hanyang University College of Medicine, Seoul 04763, Republic of Korea; joeguy@hanmail.net; 4 Department of Medicine, College of Medicine, Hanyang University, Seoul 04763, Republic of Korea; yeun0148@hanyang.ac.kr; 5 Biostatistics Lab, Medical Research Collaborating Center, Hanyang University, Seoul 04763, Republic of Korea; 6 Department of Thoracic and Cardiovascular Surgery, Hanyang University Seoul Hospital, Hanyang University College of Medicine, Seoul 04763, Republic of Korea; rgo38@naver.com; 7 Department of Thoracic and Cardiovascular Surgery, Korea University Anam Hospital, Korea University College of Medicine, Seoul 02841, Republic of Korea; fibrillary@gmail.com (Y.S.); heartistcs@korea.ac.kr (J.S.J.); 8 Division of Cardiac Surgery, Department of Surgery, Johns Hopkins Hospital, Johns Hopkins University School of Medicine, Baltimore, MD 21287, USA; jkang71@jh.edu; 9 Division of Neurosciences Critical Care, Department of Neurology, Neurosurgery, Anesthesiology and Critical Care Medicine, Johns Hopkins Hospital, Johns Hopkins University School of Medicine, Baltimore, MD 21287, USA

**Keywords:** extracorporeal life support, extracorporeal cardiopulmonary resuscitation, neuron-specific enolase, neurologic outcome, mortality

## Abstract

**Background:** Neuron-specific enolase (NSE) has traditionally been used as a biomarker to predict neurologic outcomes after cardiac arrest. This study aimed to evaluate the utility of NSE in predicting neurologic outcomes in patients undergoing extracorporeal cardiopulmonary resuscitation (ECPR). **Methods:** This observational cohort study included 47 consecutive adult ECPR patients (median age, 59.0 years; 74.5% males) treated between January 2018 and December 2021 at a tertiary extracorporeal life support center. The primary outcome was a poor neurologic outcome, defined as a Cerebral Performance Category score of 3–5 at hospital discharge. **Results:** Twelve (25.5%) patients had abnormal findings on computed tomography of the brain. A poor neurologic outcome was demonstrated in 22 (46.8%) patients. The NSE level at 72 h after ECPR showed the best prediction power for a poor neurologic outcome compared with NSE at 24 and 48 h. A cutoff value exceeding 61.9 μg/L for NSE at 72 h yielded an area under the curve (AUC) of 0.791 for predicting poor neurologic outcomes and exceeding 62.1 μg/L with an AUC of 0.838 for 30-day mortality. **Conclusions:** NSE levels at 72 h after ECPR appear to be a reliable biomarker for predicting poor neurologic outcomes and 30-day mortality in ECPR patients.

## 1. Introduction

Despite improvements in survival following extracorporeal cardiopulmonary resuscitation (ECPR), devastating neurologic injuries and poor outcomes remain common [1,2,3,4]. Prior studies have shown a high incidence of acute brain injury (ABI) in extracorporeal life support (ECLS) patients, including hypoxic-ischemic brain injury, brain death, strokes, and seizures [3,4]. Despite the common occurrence of ABI, sparse data exist on the utility of diagnostic tools such as plasma biomarkers for ABI in predicting neurologic outcomes in ECLS patients [5,6,7].

Neuron-specific enolase (NSE) has been used as a biomarker for predicting ABI because it is an enzyme found in high concentrations in neurons and neuroendocrine cells [7,8]. The pathway involving NSE as a biomarker can be summarized as follows: Neuronal injury or neuroendocrine cell damage can occur due to cardiac arrest. Damaged cells release NSE into the extracellular space, from where it enters the bloodstream and cerebrospinal fluid. NSE then circulates in the blood, where it can be detected and measured. Blood or cerebrospinal fluid samples are collected from the patient, and NSE levels are quantified using immunoassays. Elevated NSE levels are interpreted as indicative of the severity of neuronal damage [5,6,7,8].

Schoerkhuber et al. investigated the time course of serum NSE in patients resuscitated from cardiac arrest without ECLS application [9]. However, evidence on the use of NSE in ECLS is limited, with insufficient information on the optimal timing for obtaining NSE measurements, their kinetics, the impact of the ECLS circuit on NSE values, and cutoff value that can reliably predict favorable neurologic outcomes [5,6,7]. For instance, Floerchinger et al. monitored NSE levels within 24 h of ECLS implantation with a small sample size [5]. In Reuter’s study, although blood samples were collected at 24 and 72 h after ECLS initiation and neurologic prognosis was classified using the modified Rankin Scale, neuroimaging was conducted within 28 days following ECLS initiation and was not standard practice during the acute phase [6].

Given the limitation in prior data, this study aimed to determine the optimal timing for NSE measurement and establish a cutoff value for predicting neurologic outcomes in ECPR patients.

## 2. Materials and Methods

### 2.1. Study Patients

A retrospective single-center study was conducted in the intensive care unit (ICU) at Hanyang University Seoul Hospital, focusing on patients undergoing ECPR between January 2018 and December 2021. The study population comprised adult patients (≥18 years) who received cardiopulmonary resuscitation (CPR) for refractory in-hospital cardiac arrest (IHCA) and out-of-hospital cardiac arrest (OHCA). The exclusion criteria were as follows: (1) patients who died within 24 h of ECLS initiation, and (2) patients with missing NSE data.

### 2.2. ECPR Organization

Since the on-scene ECPR system has not been established in South Korea, all the patients with OHCA were immediately transported to the hospital via ambulance and received ECPR in the hospital’s emergency room upon arrival.

In our institution, the criteria for ECLS implantation included patients of all ages, encompassing those both under and over 80 years old. In actual clinical practice, while the patient’s age is important, physical condition/functional status, such as mobility before cardiac arrest, is more critical. Therefore, in two cases within our cohort, ECLS insertion was performed on patients over 80 years of age who had excellent functional status. For patients with OHCA, if the CPR time before hospital arrival (pre-hospital) exceeded 40 min, ECLS insertion was not performed. Additionally, our institution adhered to the principle that the total CPR time should not exceed 60 min. ECLS insertion was not performed in cases with pre-existing neurological deficits, but it was actively pursued in instances of witnessed cardiac arrest in patients who were originally alert.

For patients with a total CPR time of more than 30 min, target temperature management was considered immediately after ECPR. The goal was to achieve a core temperature of 34 degrees Celsius as soon as possible. The mean blood pressure was maintained between 65–75 mmHg, with efforts made to adjust it and maintain the pulsatility of the patient’s heart using a small dose of vasoactive drugs.

### 2.3. NSE Level Measurement and Brain Computed Tomography

Blood samples for NSE measurement were meticulously collected at precisely 24, 48, and 72 h following ECPR. Blood samples were obtained from the arterial line and transferred to a vacutainer ethylenediaminetetraacetic acid tube using a 17-gauge needle to minimize hemolysis. At the time of ECLS insertion, initial measurements of arterial pH, lactate, creatinine, and troponin-I levels were recorded. Additionally, a computed tomography (CT) scan of the brain was typically performed within 6 h after ECLS initiation.

### 2.4. Primary and Secondary Outcomes

A retrospective review of medical charts was conducted to assess the outcomes of the patients. The primary outcome was the neurologic outcome at discharge. Neurologic outcomes were categorized based on Cerebral Performance Category (CPC) scores, with scores of 1 (indicating good cerebral performance) and 2 (representing moderate cerebral disability) considered good neurologic outcomes. Conversely, scores of 3 (representing severe cerebral disability) to 5 (indicating brain death) were categorized as poor neurologic outcomes [10]. The secondary outcome analyzed was 30-day mortality.

### 2.5. Statistical Analysis

Continuous variables were presented as medians with interquartile ranges (IQRs) if skewed, as assessed using the Kolmogorov–Smirnov test. The Wilcoxon rank-sum test was used to compare the continuous variables. The categorical variables were expressed as frequencies and percentages and compared using the Chi-squared test or Fisher’s exact test, as appropriate. A box plot analysis was utilized to illustrate the distribution of numerical data and compare the median values across different groups. Additionally, a receiver operating characteristic (ROC) analysis was performed to evaluate the predictive value of NSE levels measured at 24, 48, and 72h after ECPR for both poor neurologic outcomes at discharge and 30-day mortality. Logistic regression modeling was utilized to calculate odds ratios and corresponding 95% confidence intervals to identify risk factors associated with poor neurologic outcomes. Adjustments were made for CPR time, arterial pH at the time of ECLS insertion, lactate level at the time of ECLS insertion, serum creatinine level at 24h after ECPR, and NSE level at 72h after ECPR over 61.9 ug/L. All the statistical analyses were conducted using SAS version 9.4 (SAS Institute Inc., Cary, NC, USA).

### 2.6. Ethics

This study was approved by the Institutional Review Board (IRB) of Hanyang University Hospital (Seoul, Republic of Korea) after waiving the need for informed consent (IRB No. HYUH 2022-06-001).

## 3. Results

### 3.1. Baseline Characteristics of All Patients

**Figure 1:**
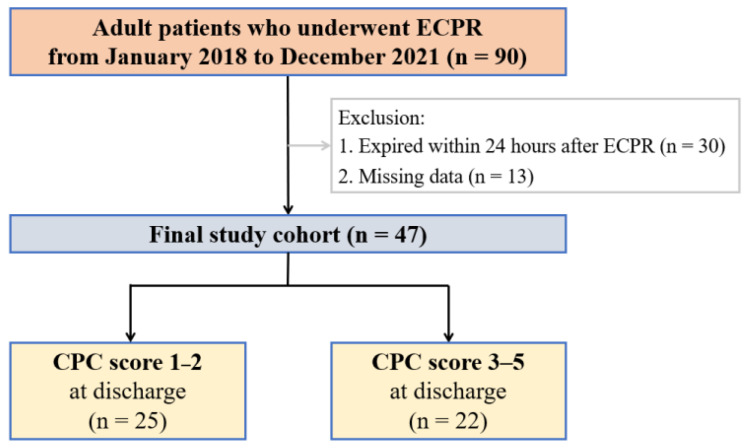
Flow diagram: extracorporeal cardiopulmonary resuscitation (ECPR), Cerebral Performance Category (CPC).

A total of 47 patients were enrolled (Figure 1), and their baseline characteristics are summarized in Table 1. The study cohort was predominantly male (74.5%), with a median age of 59.0 years (IQR, 50.0–69.0). Acute myocardial infarction was the most common cause of cardiac arrest, accounting for 59.6% of cases (*n* = 28). The median duration of CPR was 26.0 min (IQR, 12.0–41.0). The median duration of the ICU stay was 9.0 days (IQR, 5.0–14.0), while the median duration of the hospital stay was 13.0 days (IQR, 5.0–25.0). In the analysis stratified by cardiac arrest location, patients in the OHCA group were younger compared to those in the IHCA group (*p* = 0.015; Supplementary Table S1). Additionally, the OHCA group exhibited a longer total duration of CPR (*p* < 0.001) and lower arterial pH (*p* = 0.010).

The patients were divided into two groups based on their CPC score at discharge. Of 47 patients, 25 (53.2%) had a good neurologic outcome. The patients with a poor neurologic outcome had significantly higher initial lactate levels at the time of ECLS insertion compared to those with a good neurologic outcome (9.6 [IQR, 6.0–13.4] vs. 5.5 [IQR, 4.2–9.1]; *p* = 0.040).

The surviving patients at 30 days exhibited higher arterial blood pH at the time of ECLS insertion (7.2 [IQR, 7.0–7.3] vs. 7.0 [IQR, 6.9–7.2]; *p* = 0.026), lower creatinine levels at the time of ECLS insertion (1.1 [IQR, 0.9–1.6] vs. 1.4 [IQR, 1.1–2.2]; *p* = 0.048), and higher estimated glomerular filtration rate on postoperative day 1 compared to the patients who had not survived (77.0 [IQR, 48.0–97.0] vs. 57.0 [IQR, 32.0–78.5]; *p* = 0.042).

### 3.2. NSE Levels and Outcomes

**Figure 2:**
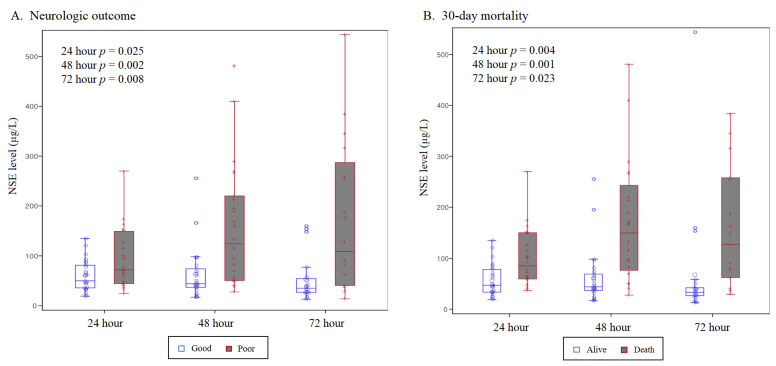
NSE level at 24, 48, and 72 h after extracorporeal cardiopulmonary resuscitation for predicting neurologic outcome and 30-day mortality. (**A**) Neurologic outcome. (**B**) 30-day mortality. Neuron-specific enolase (NSE).

The median serum levels of NSE at 24, 48, and 72 h following ECPR were 63.0 μg/L (IQR, 40.8–101.0), 64.8 μg/L (IQR, 41.1–166.0), and 41.4 μg/L (IQR, 28.6–150.6), respectively. The patients with poor neurologic outcomes consistently demonstrated significantly higher NSE levels at 24 and 48 h following ECPR compared to those with good neurologic outcomes (124.3 [IQR, 50.5–220.0] vs. 44.0 [IQR, 36.8–74.0], *p* = 0.001 at 24 h; 108.8 [IQR, 40.5–287.0] vs. 35.0 [IQR, 26.7–54.3], *p* = 0.003 at 48 h); as seen in Figure 2.

Using a threshold of an NSE level greater than 25.0 μg/L at 72 h after ECPR, the sensitivity was found to be 73.3% for predicting a poor neurologic outcome and 80.0% for predicting 30-day mortality (Table 2). Furthermore, using a threshold of NSE level greater than 75.0 μg/L at 72 h after ECPR, the specificity was 100% for both predicting a poor neurologic outcome and 30-day mortality.

**Figure 3:**
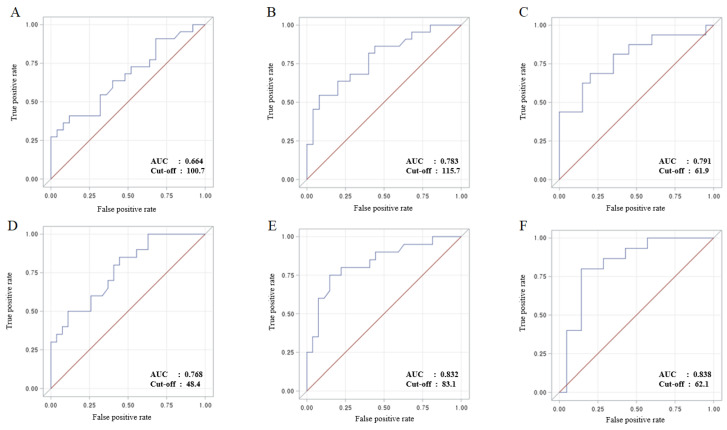
Receiver operating characteristic curves for predicting neurologic outcomes and 30-day mortality at 24, 48, and 72 h after ECPR. (**A**) NSE levels at 24 h after ECPR for predicting neurologic outcomes. (**B**) NSE levels at 48 h after ECPR for predicting neurologic outcomes. (**C**) NSE levels at 72 h after ECPR for predicting neurologic outcomes. (**D**) NSE levels at 24 h after ECPR for predicting 30-day mortality. (**E**) NSE levels at 48 h after ECPR for predicting 30-day mortality. (**F**) NSE levels at 72 h after ECPR for predicting 30-day mortality. Extracorporeal cardiopulmonary resuscitation (ECPR), neuron-specific enolase (NSE).

An ROC curve was used to determine the optimal NSE cutoff values for predicting poor neurologic outcomes and 30-day mortality (Figure 3). The area under the curve (AUC) values for NSE levels at 24, 48, and 72 h after ECPR in predicting poor neurologic outcomes were 0.664, 0.783, and 0.791, respectively. The corresponding cutoff points were determined to be 100.7 μg/L, 115.7 μg/L, and 61.9 μg/L. For predicting 30-day mortality, the AUC values for NSE levels at 24, 48, and 72 h after ECPR were 0.768, 0.832, and 0.838, respectively. The corresponding cutoff points were 48.4 μg/L, 83.0 μg/L, and 62.1 μg/L. Notably, the analysis indicated that the time point with the highest AUC value for NSE measurement was 72 h after ECPR, with cutoff points of 61.9 μg/L for predicting poor neurologic outcomes and 62.1 μg/L for predicting 30-day mortality.

In an exploratory analysis, NSE levels at 24, 48, and 72 h after ECPR were examined based on the findings of brain CT scans (Supplementary Figure S1). The patients who had abnormal findings on a brain CT (*n* = 12, 25.5%), such as hypoxic-ischemic brain injury (*n* = 7, 14.9%), ischemic stroke (*n* = 4, 8.5%), and intracranial hemorrhage (*n* = 1, 2.1%), consistently exhibited significantly higher NSE levels at all time points compared to those with normal findings (118.0 [IQR, 71.9–142.0] vs. 47.5 [IQR, 35.7–68.5], *p* = 0.002 at 24 h; 178.8 [IQR, 88.1–216.5] vs. 44.0 [IQR, 37.1–96.7], *p* = 0.002 at 48 h; 140.3 [IQR, 62.1–345.2] vs. 39.1 [IQR, 26.7–77.2], *p* = 0.003 at 72 h). In the comparison, among patients with different abnormal findings on brain CT (hypoxic-ischemic brain injury vs. ischemic stroke), NSE levels at 24, 48, and 72 h after ECPR did not show significant differences (115.0 [IQR, 72.7–149.0] vs. 127.9 [IQR, 77.3–202.5], *p* = 0.572 at 24 h; 168.5 [IQR, 95.0–213.0] vs. 225.3 [IQR, 138.1–260.8], *p* = 0.163 at 48 h; 127.3 [IQR, 90.2–258.0] vs. 268.6 [IQR, 101.7–464.0], *p* = 0.235 at 72 h); see Supplementary Figure S2.

### 3.3. Risk Factors for Poor Neurologic Outcomes

Univariable and multivariable analyses were performed to identify the risk factors associated with predicting poor neurologic outcomes and 30-day mortality, as presented in Supplementary Table S2. The covariates considered in these analyses included CPR time, arterial pH at the ECLS insertion time, lactate level at the ECLS insertion time, serum creatinine level at 24 h after ECPR, and NSE level at 72 h after ECPR over 61.9 ug/L. The NSE level exceeding 61.9 ug/L measured at 72 h was the only significant risk factor for predicting poor neurologic outcomes in both the univariable and multivariable analyses (*p* = 0.005 and *p* = 0.019, respectively).

## 4. Discussion

In this study, we found that the NSE level at 72 h after ECPR was a significant predictor of poor neurologic outcomes in ECPR patients, with a cutoff value of 61.9 μg/L and an AUC value of 0.791, respectively. Hemolysis, which frequently occurs in ECLS patients, can lead to elevations in serum NSE levels, even when no neuronal damage is apparent [6]. We attempted to minimize hemolysis, and any samples showing signs of hemolysis were excluded from the analysis. Additionally, we found that the NSE level at 72 h was useful in predicting 30-day mortality in ECPR patients, with a cutoff value of 62.1 μg/L and an AUC value of 0.838, respectively.

Among the patients included in our study, 46.8% (22/47) experienced poor neurologic outcomes. This percentage is slightly higher than those of previous studies, which reported proportions ranging from 30.7% to 38% [5,7]. However, we considered CPC score 3 as a poor neurologic outcome, whereas other studies categorized CPC scores 1–3 as good and 4–5 as poor outcomes [11]. It is worth noting that patients with poor neurologic outcomes had shorter hospital stays due to early mortality.

Recent ECLS randomized controlled trials highlighted the importance of neurological complications on mortality/survival [12,13], Thus, standardized neuroimaging and neurological diagnosis are crucial for predicting neurologic outcomes in patients after resuscitation and ECLS [5]. In our study, 39 (83.0%) patients had brain CT scans as clinically indicated. Among 12 (25.5%) of the patients who displayed abnormal brain CT scan results, including conditions such as hypoxic-ischemic brain injury, ischemic stroke, and intracranial hemorrhage, there was a consistent and significant elevation in their NSE levels at 24, 48, and 72 h after ECPR compared to those with normal findings. However, due to the time constraints, the initial brain CT scans in our study were typically performed within 6 h after ECLS initiation. This timing may not have been sufficient to capture ABI, as early ischemic changes are often missed in CT scans [14,15]. Despite this, given the many challenges in transporting ECLS patients for neuroimaging, serum biomarkers like NSE are highly convenient and valuable tools due to their safety and ease of use [16].

Furthermore, to overcome the incompatibility of examinations in a magnetic resonance imaging (MRI) room due to the metallic properties of the console and cannula, Cho et al. reported initial experiences involved the use of a portable brain MRI at the ICU bedside to assess ABI, focusing on safety and feasibility [17].

Combining the results of these examinations with our findings could enhance the accuracy of neurologic outcome predictions. By incorporating multiple evaluation tools, healthcare providers can obtain a more comprehensive understanding of the patient’s neurologic status and make more informed decisions regarding treatment and care. It is important to note that while these evaluation tools provide valuable insights, the interpretation of results should be done in conjunction with other clinical factors and individual patient characteristics. A multimodal approach that considers a combination of clinical assessments, biomarkers, and imaging modalities should be taken to evaluate neurologic outcomes in patients undergoing ECPR [18]. The NSE level at 72 h after ECPR was the best predictor of a poor neurological outcome in our study. Therefore, NSE should be used as an adjunct in a comprehensive assessment of neurological status in ECPR patients to enhance prognostication.

The findings of our study are consistent with Schoerkhuber’s research, demonstrating the most significant difference in NSE levels at 72 h after cardiac arrest [9]. However, Schoerkhuber’s study focused solely on patients who received conventional CPR and only included those with a return of spontaneous circulation, thereby investigating only survivors. As a result, many patients who could have survived with ECPR were not considered. Therefore, our study can be viewed as providing more comprehensive real-world data. Furthermore, our study provides a threshold analysis for NSE cutoff values accounting for having an ECLS circuit, which could alter the kinetics of NSE.

In our study, we found that elevated lactate levels and low arterial pH at the time of ECPR, as well as elevated creatinine levels at 24 h after ECPR, are associated with poor neurologic outcomes. Previous studies have reported the association between elevated lactate levels and mortality [19,20]. More recently, several studies have demonstrated the relationship between elevated serum lactate levels and poor neurologic outcomes, specifically in patients undergoing ECLS [21,22,23]. Compared to these previous studies [21,22,23], our study, although conducted at a single center with a small sample size, included both OHCA and IHCA patients. We investigated various variables such as pH and creatinine and observed NSE levels serially.

Interestingly, a few studies have suggested that elevated lactate levels in the cerebrospinal fluid are associated with delayed cerebral ischemia in patients with an intracranial hemorrhage, which could predict poor neurologic outcomes [24,25]. As lactate can cross the blood–brain barrier, its level can indicate cerebral metabolic derangements and subsequent ABIs [26]. Therefore, monitoring serum lactate levels in patients undergoing ECLS could provide valuable information about their neurologic prognosis. Furthermore, our study confirms the relationship between acidosis and poor outcomes, as compromised tissue perfusion leading to acidosis can contribute to multiple organ failures [27]. This finding aligns with previous reports that have consistently linked acidosis to unfavorable outcomes [27,28,29]. Our study highlights the significance of aggressively addressing and correcting acid–base imbalances in patients undergoing ECLS. Taken together, our findings suggest that elevated lactate levels and acidosis are valuable predictors of both neurologic outcomes and 30-day mortality in patients undergoing ECLS.

One of the interesting findings in our research is that, despite OHCA patients having a CPR time nearly twice as long as that of IHCA patients, the 30-day mortality is not significantly different between the two groups. Our country has an excellent emergency patient transport system, and our institution is a tertiary emergency medical center with close coordination between ambulances and the emergency room. Although ECLS insertion is not performed on-scene outside the hospital, preparations are made before the patient’s arrival at the hospital emergency room. When patients undergoing CPR arrive, the on-call team is activated, and ECLS insertion is performed promptly, ensuring the system is well-established to avoid wasting time. In contrast, for patients with IHCA, the performance is acceptable in the ICU or cath lab. However, for patients who arrest in a general ward, the time required for ICU transfer and potential delays are not negligible, which likely contributed to these results. A previous paper reported that the short-term outcomes for patients with OHCA in our country were more favorable compared to the international data [30].

As shown in Table 1, 44 (93.6%) patients in the total cohort had a cardiac-related diagnosis as the cause of cardiac arrest. Of the remaining three patients, two had electrolyte imbalances, and one had septic shock. The cardiac-related diagnoses were further subdivided, resulting in small numbers for each diagnosis category, making direct outcome comparisons difficult. Among the 31 patients with coronary artery disease (e.g., acute myocardial infarction and ischemic cardiomyopathy), 16 (51.6%) had a good neurologic outcome, while 15 (48.4%) had a poor neurologic outcome.

While the standardization of prognostic studies improved following the publication of the Transparent Reporting of a multivariable prediction model for Individual Prognosis Or Diagnosis guidelines in 2015, these guidelines do not address factors that reflect the impact of the self-fulfilling prophecy bias [31,32]. Among our cohort, there were only four patients identified as cases of self-fulfilling prophecy, all of whom were classified under the poor neurologic outcome group and died due to ECLS weaning failure. These patients died following the withholding of life-sustaining therapies based on their poor prognoses. However, even without the withholding of therapies, these patients would have had a poor outcome, indicating that the prognosis did not alter the outcome. These results were all true positives, and we were able to minimize bias related to the self-fulfilling prophecy [33]. As only four patients underwent a withdrawal of life-sustaining therapy, the concern for self-fulfilling prophecy is much lesser than in other studies.

### Strengths and Limitations of This Study

The strengths of this study include the extensive clinical information obtained through brain CT scans in many patients. We investigated not only NSE but also other factors influencing poor neurologic outcomes, such as elevated lactate levels and low arterial pH at the time of ECLS insertion. Furthermore, our study examined the serial trend of NSE and aimed to establish a standard for clinical practice by suggesting a cut-off value at an appropriate time point.

However, this study had several limitations. First, it was a single-center, retrospective study, which may limit the generalizability of the findings to other settings or populations. Future studies with larger sample sizes and multi-center designs are needed to validate our results and enhance their applicability. Second, the origin of NSE is uncertain, as it can potentially be released from sources other than the brain, such as from hemolysis. Although we attempted to minimize hemolysis during ECLS insertion and sample collection, it was difficult to assert that these efforts entirely eliminated hemolysis. Future research should incorporate methods to more rigorously control for hemolysis and other potential confounders and potentially add plasma biomarkers that are not influenced by hemolysis. Third, we excluded patients who died within 24 h of ECLS initiation to focus on neurologic outcomes, which may introduce survivorship bias. It is important to know that patients who died within 24 h of cannulation did not have sufficient time to be given a neurologic diagnosis and often, it is difficult to get consent for obtaining plasma samples if patients die too early. Therefore, there were also limitations of including these patients that led to a different type of bias. Future analyses will include these patients to provide a comprehensive evaluation of NSE’s predictive value. Fourth, brain CT scans were typically performed within 6 h after ECLS initiation, and this might miss early ischemic changes in the brain. However, we also would like to highlight that performing brain CT scans within 6 h as a protocol is not routinely done in many centers due to the difficulty with transportation and logistics/resource issues, so our study offers the advantage of having scans in all of these patients. In follow-up studies, we will implement a strategy to perform CT scans before hospital discharge.

## 5. Conclusions

NSE levels at 72 h after ECPR appear to be a reliable biomarker for predicting poor neurologic outcomes and 30-day mortality in ECPR patients. The most accurate NSE cutoff value was 61.9 μg/L for predicting poor neurologic outcome and 62.1 μg/L for predicting 30-day mortality.

## Figures and Tables

**Table 1:** Baseline characteristics of all the patients.

	All Patients (*n* = 47)	CPC Score 1–2 (*n* = 25)	CPC Score 3–5 (*n* = 22)	*p*-Value	Alive (*n* = 27)	Death (*n* = 20)	*p*-Value
Age	59.0 (50.0–69.0)	58.0 (51.0–64.0)	64.5 (49.0–75.0)	0.182	59.0 (51.0–67.0)	62.0 (48.5–69.0)	0.870
Male	35 (74.5)	19 (76.0)	16 (72.7)	>0.999	21 (77.8)	14 (70.0)	0.737
Cause of cardiac arrest				0.565			0.436
Acute MI	28 (59.6)	13 (52.0)	15 (68.2)		15 (55.6)	13 (65.0)	
ICMP	3 (6.4)	3 (12.0)	0 (0.0)		3 (11.1)	0 (0.0)	
DCMP	2 (4.3)	1 (4.0)	1 (4.6)		0 (0.0)	2 (10.0)	
Acute myocarditis	4 (8.5)	3 (12.0)	1 (4.6)		3 (11.1)	1 (5.0)	
PTE	2 (4.3)	1 (4.0)	1 (4.6)		1 (3.7)	1 (5.0)	
Infective endocarditis	1 (2.1)	0 (0.0)	1 (4.6)		0 (0.0)	1 (5.0)	
SCMP	2 (4.3)	1 (4.0)	1 (4.6)		1 (3.7)	1 (5.0)	
Fatal arrhythmia	2 (4.3)	2 (8.0)	0 (0.0)		2 (7.4)	0 (0.0)	
Others	3 (6.4)	1 (4.0)	2 (9.1)		2 (7.4)	1 (5.0)	
Location of cardiac arrest				0.556			0.152
In-hospital	27 (57.5)	13 (52.0)	14 (63.6)		13 (48.2)	14 (70.0)	
Out-of-hospital	20 (42.6)	12 (48.0)	8 (36.4)		14 (51.9)	6 (30.0)	
CPR time							
Total	26.0 (12.0–41.0)	32.0 (10.0–40.0)	25.0 (17.0–44.0)	0.624	33.0 (10.0–40.0)	25.0 (16.0–42.5)	0.949
Pre-hospital (OHCA, *n* = 20)	19.5 (9.5–29.0)	18.0 (9.0–30.0)	26.0 (9.5–28.0)	0.054	19.5 (10.0–29.0)	18.0 (9.0–27.0)	0.109
In-hospital	21.0 (9.0–32.0)	15.0 (6.0–32.0)	23.5 (15.0–31.0)	0.267	15.0 (6.0–32.0)	23.5 (16.0–31.0)	0.249
pH							
ECLS insertion time	7.1 (7.0–7.3)	7.2 (7.1–7.3)	7.0 (6.9–7.3)	0.080	7.2 (7.0–7.3)	7.0 (6.9–7.2)	0.026
POD#1	7.4 (7.3–7.5)	7.4 (7.3–7.5)	7.4 (7.3–7.5)	0.529	7.4 (7.3–7.5)	7.4 (7.3–7.4)	0.132
Lactate							
ECLS insertion time	7.4 (4.6–12.1)	5.5 (4.2–9.1)	9.6 (6.0–13.4)	0.040	5.5 (4.0–8.9)	9.6 (6.4–12.7)	0.041
POD#1	5.7 (2.8–9.0)	4.6 (2.4–6.9)	7.6 (5.4–9.6)	0.064	4.6 (2.4–6.9)	8.1 (5.5–9.6)	0.046
Creatinine							
ECLS insertion time	1.2 (0.9–1.7)	1.2 (0.9–1.6)	1.3 (1.1–1.7)	0.190	1.1 (0.9–1.6)	1.4 (1.1–2.2)	0.048
POD#1	1.1 (0.9–1.8)	1.0 (0.9–1.4)	1.2 (0.9–1.9)	0.147	1.0 (0.9–1.4)	1.4 (1.0–2.0)	0.083
eGFR							
ECLS insertion time	64.0 (34.0–83.0)	72.0 (50.0–91.0)	55.0 (28.0–68.0)	0.086	72.0 (50.0–91.0)	53.0 (27.0–66.0)	0.031
POD#1	71.0 (40.0–91.0)	78.0 (48.0–97.0)	58.5 (31.0–75.0)	0.064	77.0 (48.0–97.0)	57.0 (32.0–78.5)	0.042
Troponin-I							
ECLS insertion time	0.2 (0.1–1.0)	0.3 (0.1–1.7)	0.1 (0.1–0.8)	0.668	0.3 (0.1–2.3)	0.2 (0.1–0.7)	0.629
POD#1	46.0 (1.8–50.0)	46.0 (2.2–50.0)	43.5 (1.8–50.0)	0.930	46.0 (0.8–50.0)	43.5 (2.2–50.0)	0.833
Brain CT (*n* = 39)				0.002			0.003
Normal	27 (69.2)	18 (90.0)	9 (47.4)		18 (81.8)	9 (52.9)	
Hypoxic-ischemic brain injury	7 (18.0)	0 (0.0)	7 (36.8)		0 (0.0)	7 (41.2)	
Ischemic stroke	4 (10.3)	2 (10.0)	2 (10.5)		3 (13.6)	1 (5.9)	
Intracranial hemorrhage	1 (2.6)	0 (0.0)	1 (5.3)		1 (4.6)	0 (0.0)	
Optic nerve diameter (*n* = 39)	5.4 (4.9–5.8)	5.5 (5.0–5.9)	5.3 (4.9–5.6)	0.285	5.4 (5.0–5.8)	5.3 (4.9–5.7)	0.788
NSE							
POD#1	63.0 (40.8–101.0)	49.9 (35.7–81.2)	71.9 (44.2–149.0)	0.056	47.0 (33.6–78.1)	85.2 (59.4–150.0)	0.002
POD#2	64.8 (41.1–166.0)	44.0 (36.8–74.0)	124.3 (50.5–220.0)	0.001	44.0 (36.8–69.0)	149.5 (76.4–243.0)	<0.001
POD#3 (*n* = 36)	41.4 (28.6–150.6)	35.0 (26.7–54.3)	108.8 (40.5–287.0)	0.003	33.1 (26.6–42.1)	127.3 (62.1–258.0)	<0.001
ICU stay	9.0 (5.0–14.0)	12.0 (8.0–14.0)	6.0 (4.0–14.0)	0.016	12.0 (7.0–17.0)	7.0 (4.0–12.5)	0.026
Hospital stay	13.0 (5.0–25.0)	20.0 (8.0–29.0)	7.0 (4.0–14.0)	0.001	20.0 (8.0–30.0)	7.0 (4.0–12.5)	0.001

Continuous variables are presented as medians (interquartile ranges). Categorical variables are presented as frequencies and proportions. Cerebral Performance Category (CPC), myocardial infarction (MI), ischemic cardiomyopathy (ICMP), dilated cardiomyopathy (DCMP), pulmonary thromboembolism (PTE), stress-induced cardiomyopathy (SCMP), cardiopulmonary resuscitation (CPR), out-of-hospital cardiac arrest (OHCA), extracorporeal life support (ECLS), estimated glomerular filtration rate (eGFR), postoperative day (POD), neuron-specific enolase (NSE), intensive care unit (ICU).

**Table 2:** Diagnostic accuracy of neuron-specific enolase level predicting a poor neurologic outcome (cerebral performance category score 3–5) during hospitalization and 30-day mortality.

Variable	CPC Score 3–5 at Discharge		Variable	30-Day Mortality	
	Sensitivity (95% CI)	Specificity (95% CI)		Sensitivity (95% CI)	Specificity (95% CI)
NSE level at 24 h after ECPR			NSE level at 24 h after ECPR		
NSE > 25	48.9 (28.0–69.8)	87.0 (73.8–100)	NSE > 25	50.0 (28.1–71.9)	87.5 (75.0–100.0)
NSE > 50	40.0 (19.5–60.5)	100.0 (100.0–100.0)	NSE > 50	37.5 (16.3–58.7)	100.0 (100.0–100.0)
NSE > 75	60.0 (39.5–80.5)	100.0 (100.0–100.0)	NSE > 75	54.6 (32.7–76.4)	100.0 (100.0–100.0)
NSE level at 48 h after ECPR			NSE level at 48 h after ECPR		
NSE > 25	54.5 (33.7–75.4)	90.0 (80.9–99.1)	NSE > 25	75.0 (56.0–94.0)	81.8 (67.3–96.4)
NSE > 50	66.7 (47.0–86.4)	80.0 (64.3–95.7)	NSE > 50	70.6 (50.6–90.6)	81.8 (67.3–96.4)
NSE > 75	71.4 (52.5–90.3)	83.3 (68.7–98.0)	NSE > 75	80.0 (62.5–97.5)	60.0 (41.5–78.5)
NSE level at 72 h after ECPR			NSE level at 72 h after ECPR		
NSE > 25	73.3 (54.9–91.8)	75.0 (58.0–92.0)	NSE > 25	80.0 (62.5–97.5)	81.3 (66.5–96.0)
NSE > 50	63.6 (43.5–83.7)	100.0 (100.0–100.0)	NSE > 50	100.0 (100.0–100.0)	40.0 (21.5–58.5)
NSE > 75	70.0 (50.9–89.2)	100.0 (100.0–100.0)	NSE > 75	45.5 (23.6–67.3)	100.0 (100.0–100.0)

Cerebral Performance Category (CPC), confidence interval (CI), neuron-specific enolase (NSE), extracorporeal cardiopulmonary resuscitation (ECPR).

## Data Availability

The data supporting the findings of this study are available from the corresponding author (J.H.L.) upon reasonable request.

## Supplementary Materials

The following supporting information can be downloaded at: https://www.mdpi.com/article/10.3390/jcm13144135/s1, Supplementary Figure S1. NSE levels at 24, 48, and 72 hours after extracorporeal cardiopulmonary resuscitation based on the brain computed tomography findings. Supplementary Figure S2. NSE levels at 24, 48, and 72 hours after extracorporeal cardiopulmonary resuscitation based on abnormal findings (i.e. hypoxic-ischemic brain injury, ischemic stroke) on brain computed tomography. Supplementary Table S1. Baseline characteristics of all patients according to where the cardiac arrest occurred. Supplementary Table S2. Univariable and multivariable analyses of poor neurologic outcomes.

## Abbreviations

ABI	acute brain injury
AUC	area under the curve
CPC	Cerebral Performance Category
CPR	cardiopulmonary resuscitation
CT	computed tomography
ECLS	extracorporeal life support
ECPR	extracorporeal cardiopulmonary resuscitation
ICU	intensive care unit
IHCA	in-hospital cardiac arrest
MRI	magnetic resonance imaging
NSE	neuron-specific enolase
OHCA	out-of-hospital cardiac arrest
ROC	receiver operating curve
