# Supplementary material for: Neuron-Specific Enolase as a Predictor of Neurologic Outcomes in Extracorporeal Cardiopulmonary Resuscitation Patients
Source: J Clin Med. 2024 Jul 15;13(14):4135. doi: 10.3390/jcm13144135 (PMC11277770; doi:10.3390/jcm13144135)
Supplement: Supplementary file 1 [file jcm-13-04135-s001.zip › Supplementary Table S2.pdf]

Supplementary Table S2. Univariable and multivariable analyses of poor neurologic outcomes.

	Univariable analysis OR (95% CI)	*p* value	Multivariable analysis OR (95% CI)	*p* value
CPR time	1.004 (0.976–1.033)	0.777	0.980 (0.936–1.025)	0.374
Arterial pH at the ECLS insertion time	0.049 (0.002–1.336)	0.074	18.333 (0.020– > 999.999)	0.403
Lactate level at the ECLS insertion time	1.166 (1.009–1.348)	0.038	1.305 (0.977–1.744)	0.072
Creatinine level at 24 hours after ECPR	1.183 (0.740–1.891)	0.483	1.030 (0.510–2.082)	0.934
NSE level at 72 hours after ECPR over 61.9 ug/L	8.800 (1.920–40.336)	0.005	8.407 (1.413–50.028)	0.019

ORs and 95% CIs were calculated using logistic regression modeling.

Odds ratio (OR), confidence interval (CI), cardiopulmonary resuscitation (CPR), extracorporeal life support (ECLS), extracorporeal cardiopulmonary resuscitation (ECPR), neuron-specific enolase (NSE).
