# Supplementary material for: Neuron-Specific Enolase as a Predictor of Neurologic Outcomes in Extracorporeal Cardiopulmonary Resuscitation Patients
Source: J Clin Med. 2024 Jul 15;13(14):4135. doi: 10.3390/jcm13144135 (PMC11277770; doi:10.3390/jcm13144135)
Supplement: Supplementary file 1 [file jcm-13-04135-s001.zip › Supplementary Table S1.pdf]

Supplementary Table S1. Baseline characteristics of all patients according to where the cardiac arrest occurred.

	IHCA (n = 27)	OHCA (n = 20)	*p* value
Age	64.0 (51.0–72.0)	54.0 (48.0–59.5)	0.015
Male	21 (77.8)	14 (70.0)	0.737
Cause of cardiac arrest			0.120
Acute MI	18 (66.7)	10 (50.0)	
ICMP	0 (0.0)	3 (15.0)	
DCMP	2 (7.4)	0 (0.0)	
Acute myocarditis	2 (7.4)	2 (10.0)	
PTE	1 (3.7)	1 (5.0)	
Infective endocarditis	0 (0.0)	1 (5.0)	
SCMP	2 (7.4)	0 (0.0)	
Fatal arrhythmia	0 (0.0)	2 (10.0)	
Others	2 (7.4)	1 (5.0)	
CPC score			0.556
CPC score 1–2	13 (48.2)	12 (60.0)	
CPC score 3–5	14 (51.9)	8 (40.0)	
30-day mortality			0.152
Alive	13 (48.2)	14 (70.0)	
Death	14 (51.9)	6 (30.0)	
CPR time			
Total	20.0 (6.0–25.0)	42.5 (34.5–56.0)	< 0.001
Pre-hospital	-	19.5 (9.5–29.0)	-
In-hospital	20.0 (6.0–25.0)	28.5(11.5–32.5)	0.143
pH			
ECLS insertion time	7.2 (7.1–7.3)	7.0 (6.9–7.2)	0.010
POD#1	7.4 (7.3–7.5)	7.4 (7.3–7.5)	0.401
Lactate			
ECLS insertion time	6.3 (4.4–9.2)	9.9 (5.3–13.4)	0.061
POD#1	5.7 (2.3–9.6)	5.7 (3.1–8.9)	0.900
Creatinine			
ECLS insertion time	1.2 (1.0–1.7)	1.2 (0.9–1.7)	0.863
POD#1	1.1 (0.9–1.7)	1.0 (0.8–2.3)	0.739
eGFR			

ECLS insertion time	64.0 (30.0–87.0)	67.0 (47.0–82.5)	0.974
POD#1	71.0 (44.0–83.0)	72.5 (32.0–98.5)	0.561
Troponin-I			
ECLS insertion time	0.2 (0.1–3.3)	0.3 (0.1–0.8)	0.849
POD#1	50.0 (1.8–50.0)	6.1 (1.5–50.0)	0.358
ICU stay	11.0 (7.0–17.0)	8.5 (4.5–12.0)	0.185
Hospital stay	13.0 (7.0–25.0)	11.5 (4.5–23.5)	0.419

In-hospital cardiac arrest (IHCA), myocardial infarction (MI), out-of-hospital cardiac arrest (OHCA), ischemic cardiomyopathy (ICMP), dilated cardiomyopathy (DCMP), pulmonary thromboembolism (PTE), stress-induced cardiomyopathy (SCMP), Cerebral Performance Category (CPC), cardiopulmonary resuscitation (CPR), extracorporeal life support (ECLS), postoperative day (POD), estimated glomerular filtration rate (eGFR), intensive care unit (ICU).
