# Supplementary figures and images for: Neuron-Specific Enolase as a Predictor of Neurologic Outcomes in Extracorporeal Cardiopulmonary Resuscitation Patients
Source: J Clin Med. 2024 Jul 15;13(14):4135. doi: 10.3390/jcm13144135 (PMC11277770; doi:10.3390/jcm13144135)

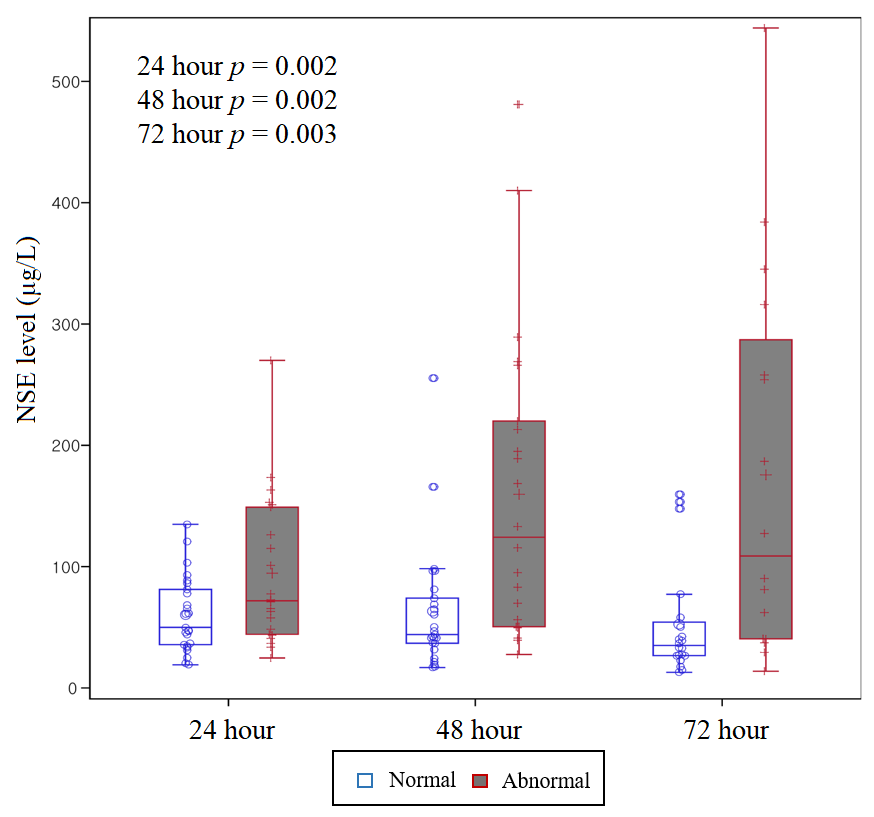

Supplement: Supplementary file 1 [file jcm-13-04135-s001.zip › Supplementary Figure S1.tif]

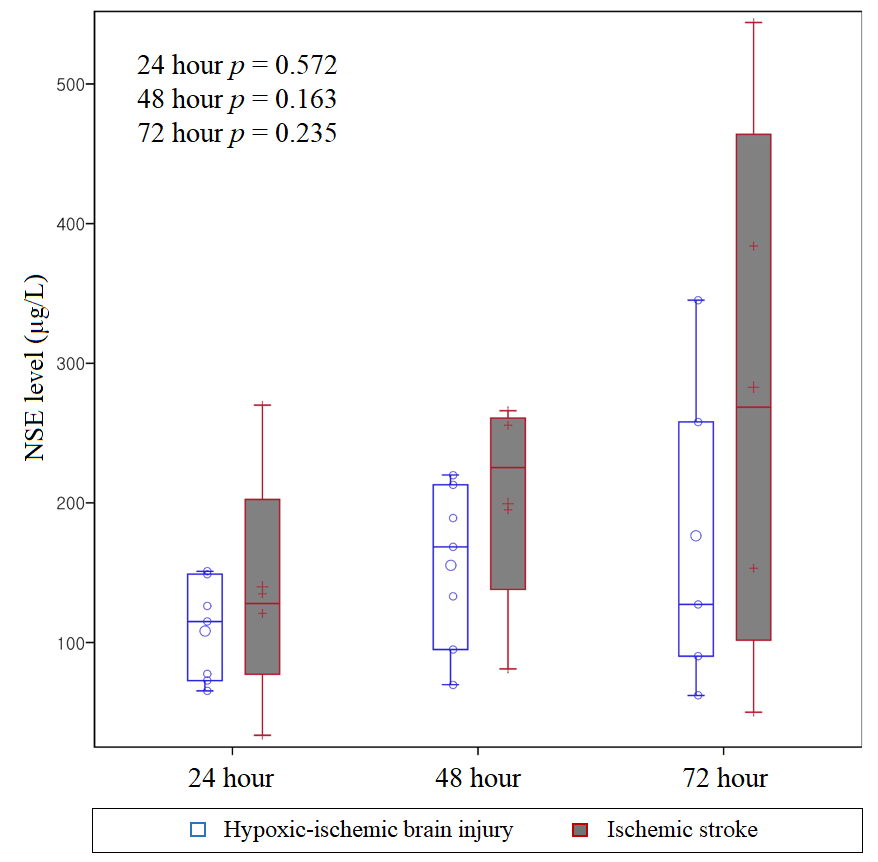

Supplement: Supplementary file 1 [file jcm-13-04135-s001.zip › Supplementary Figure S2.tif]
